# Vaccination for SARS-CoV-2 in Patients With Psoriatic Arthritis: Can Therapy Affect the Immunological Response?

**DOI:** 10.3389/fmed.2022.811829

**Published:** 2022-02-28

**Authors:** Maurizio Benucci, Arianna Damiani, Maria Infantino, Mariangela Manfredi, Barbara Lari, Valentina Grossi, Elena Biancamaria Mariotti, Alberto Corrà, Cristina Aimo, Lavinia Quintarelli, Alice Verdelli, Francesca Li Gobbi, Emiliano Antiga, Marzia Caproni

**Affiliations:** ^1^Rheumatology Unit, S. Giovanni di Dio Hospital, Azienda USL-Toscana Centro Florence, Florence, Italy; ^2^Rheumatology Unit, Department of Clinical and Experimental Medicine, University of Florence, Florence, Italy; ^3^Immunology and Allergology Laboratory, S. Giovanni di Dio Hospital, Azienda USL-Toscana Centro, Florence, Italy; ^4^Department of Health Sciences, Section of Dermatology, University of Florence, Florence, Italy; ^5^Rare Diseases Unit, Azienda USL Toscana Centro, European Reference Network-Skin Member, Department of Health Sciences, University of Florence, Florence, Italy

**Keywords:** SARS-CoV-2 vaccination, psoriatic arthritis, BNT162b2, mRNA COVID-19 vaccine, DMARDs, biologics

## Abstract

**Background:**

A few studies on vaccination in patients with rheumatic diseases, including arthritis, connective tissue diseases, vasculitis, and psoriatic arthropathy (PsA), demonstrated reduced production of neutralizing antibodies to SARS-CoV-2 Spike RBD (receptor-binding domain contained in the N-terminal of the S1 globular head region) when compared to the general population.

**Objective:**

The aim of our study was to observe whether different therapies for PsA [methotrexate, anti-TNF antibodies, soluble TNF receptor (etanercept) or IL-17 inhibitors] have a different impact on SARS-CoV-2 vaccination in a homogeneous population of patients.

**Methods:**

We enrolled 110 PsA patients in remission, assessed with Disease Activity in PSoriatic Arthritis (DAPSA). Of these: 63 were in treatment with anti-TNF-α therapy (26 etanercept, 15 certolizumab, 5 golimumab, 17 adalimumab); 37 with anti-IL17 secukinumab; 10 with methotrexate. All patients underwent vaccination for SARS-CoV-2 with mRNA BNT162b2 vaccine. Assessment of absolute and percentage lymphocyte subsets and anti-SARS-CoV-2 Spike RBD IgG antibody value 3 weeks after the second vaccine dose were performed. In addition, the serum antibody levels of 96 healthy healthcare workers (HCW) were analyzed.

**Results:**

The mean disease activity assessed with DAPSA score was 2.96 (SD = 0.60) with no significant differences between patients under different medications (*p* = 0.779). Median levels of neutralizing antibodies to SARS-CoV-2 Spike RBD were 928.00 binding antibody unit (BAU)/mL [IQR 329.25, 1632.0]; 1068.00 BAU/ml [IQR 475.00, 1632.00] in patients taking MTX, 846.00 BAU/ml [IQR 125.00, 1632.00] in patients taking etanercept, 908.00 BAU/mL [IQR 396.00, 1632.00] in patients taking anti-IL17 and 1148.00 BAU/ml [IQR 327.00, 1632.00] in patients taking TNF-α inhibitors, without statistically significant differences between these groups. Mean serum antibody level of HCW group was 1562.00 BAU/ml [IQR 975.00, 1632.00], being significantly higher than in the patient group (*p* = 0.000816). Absolute and percentage count of lymphocyte subsets were not statistically different between the subgroups under different treatments and when compared with HCW.

**Conclusions:**

As for other rheumatic diseases on immunomodulatory treatment, our data showed a reduced humoral response in PsA patients compared to the control group. However, antibody response did not significantly differ between groups treated with different medications.

## Introduction

Psoriatic arthritis (PsA) is a clinical heterogeneous, progressive and chronic inflammatory condition potentially leading to irreversible joint damage with negative impact on patient's quality of life (1–4). Many factors, including disease's subset (peripheral arthritis, axial disease, enthesitis, dactylitis, skin psoriasis, nail psoriasis), and severity, along with failure to previous lines of treatment, need to be considered when setting up a therapy for active PsA according with current guideline recommendations (5–7). Treatment options for PsA include non-biologic disease-modifying antirheumatic drugs (DMARDs) such as methotrexate, sulfasalazine, ciclosporin, and leflunomide, biologic therapies such as infliximab, golimumab, adalimumab, etanercept, certolizumab pegol, abatacept, ustekinumab, secukinumab, and ixekizumab and targeted synthetic DMARDs (i.e., apremilast and tofacitinib). These biologic and targeted therapies are used with optional concomitant DMARDs treatment. Due to immune dysregulation, PsA patients often receive corticosteroids (CS) and immunosuppressive therapies as well as other rheumatic disease. Moreover, they frequently have comorbidities such as diabetes, obesity, hypertension or may show lung or kidney involvement (8). For these reasons, patients with PsA have been included in the fragile patient's category, according to the Italian Ministry of Health, with the priority to SARS-CoV-2 vaccination, being considered a category at higher risk of developing coronavirus disease (COVID-19) with severe outcome (9, 10). The international community of rheumatologists have been focused on the effects of COVID-19 on their patients receiving different anti-rheumatic therapies. Thus, both European League Against Rheumatism (EULAR) and the American College of Rheumatology (ACR) in 2020 developed a guidance for the management of rheumatic diseases in adult patients during the COVID-19 pandemic (11, 12) but did not reach a consensus over the withholding of all the drugs at disposal, in the peri-vaccination period.

In fact, while the effect of immunosuppressive agents on the immunogenicity of other vaccines has been largely investigated, to reach a consensus over their effect on anti-SARS-CoV-2 vaccines more data are needed. Studies on the effect of vaccination in rheumatic patients with arthritis, connective tissue diseases, vasculitis and PsA have demonstrated a low level of neutralizing antibodies to SARS-CoV-2 compared to the general population (13–15). In particular, it has been reported that methotrexate impairs serological response SARS-CoV-2 vaccine-induced immunity, even in absence of significant impact on seroconversion rate (16–18), while TNF-α inhibitors seems not to affect the ability to mount a sufficient serological and cellular response to two doses of SARS-CoV-2 mRNA BNT162b2 vaccine in psoriasis patients (17). Moreover, according to recent evidence, anti-IL17 and secukinumab, in particular, do not seem to interfere significantly with seroconversion rate following mRNA SARS-CoV-2 vaccine also (15, 17, 19).

The aim of our study was to acquire more data over the impact of different therapies for PsA such as methotrexate, anti-TNF-α antibodies, soluble TNF receptor (etanercept) or IL-17 inhibitors, on SARS-CoV-2 vaccination.

## Methods

We studied 110 PsA patients enrolled at the Rheumatology Unit of the S. Giovanni di Dio Hospital (Florence) from July to October 2021. Concurrently, 96 healthy healthcare workers (HCW group) were enrolled as healthy controls. The following characteristics were considered as inclusion criteria: age above 18 years; previous administration of both first and second dose of SARS-CoV-2 BNT162b2 vaccine; stable therapy regimen from at least 12 months; PsA in clinical remission, intended as a value ≤ 4 resulted from the assessment with Disease Activity in PSoriatic Arthritis (DAPSA) score at the time of enrolment (20). Conversely, previous SARS-CoV-2 infection, concomitant systemic corticosteroid treatment and autoimmune or immunodeficiencies comorbidities were designed as exclusion criteria. All the patients included in the study were on immunomodulatory treatment and on monotherapy regimen at the time of their enrollment: 63 were in treatment with anti-TNF therapy; 37 with secukinumab (150 mg every 4 weeks); 10 with methotrexate (MTX, 10 mg weekly). Among the patients treated with anti-TNF: 26 were on etanercept (50 mg weekly), 17 on adalimumab (40 mg every 2 weeks), 15 on certolizumab pegol (200 mg/every 2 weeks), 5 on golimumab (50 mg every 4 weeks). The patients under methotrexate were told to withhold the administration of the drug 1 week after each vaccine dose, while subjects undergoing biological agents did not change their treatment schedule, as recommended by the latest available ACR COVID-19 vaccine clinical guidance at the time of enrolment (21) (version 2.0, July 2021).

All the enrolled PsA patients underwent evaluation of the lymphocyte subpopulations (CD3+, CD3+/ CD4+, CD3+/CD8+, CD4+/CD8+ ratio, CD3-/CD19+, CD3-/CD56+CD16+) by a flow cytometry analysis (FACS CANTO II, BD Biosciences) and the titer of anti-SARS-CoV-2 Spike RBD (receptor-binding domain contained in the N-terminal of the S1 globular head region) IgG antibodies (quantified by FEIA ThermoFisher, Uppsala Sweden) was also determined in both patients and HCW groups. All the mentioned analysis were conducted 3 weeks after the second vaccine injection. All patients gave their written informed consent based on the prospective nature of the study according to the Declaration of Helsinki and to the Italian legislation (Authorization of the Privacy Guarantor n.9, 12 December 2013). Local scientific ethic committee and health department examined and approved this research and the use of clinical and laboratory data of common clinical practice, in compliance with the Privacy Law, for clinical and scientific studies and publications.

### Statistical Analysis

Statistical analysis was performed using R 3.5.2 GUI 1.70 El Capitan build (7612) software. For the descriptive statistics, continuous variables were tested for normality of distribution using Shapiro- Wilk test and represented by indicating the average and standard deviation in case of normality. Non-normally distributed variables were indicated as median and interquartile range [IQR]. Categorical variables were described by frequency distribution. Parametric (One way ANOVA) or non-parametric (Kruskall Wallis) tests, as appropriate, were than performed to compare antibody levels between patients under different therapies and between patients and controls. Linear regression analysis with stepwise selection based on *p*-value was performed considering as outcome variable binding antibody unit (BAU)/ml levels and as predictors the variables concerning characteristics of patients (sex, age, DMARD, DAPSA). Correlation between BAU/ml levels and demographical variables (sex, age) was also assessed *via* linear regression analysis in the control group.

## Results

The patient cohort consisted of 71 (65%) females and 39 (35%) males with a mean age of 61.72 years (SD 12). The prevalence of several comorbidities was collected: two patients (1.8%) had previous history of myocardial infarction, while angina pectoris was reported in three cases (2.7%). Twenty-five subjects were affected by arterial hypertension (22.7%) and one case by peripheral vascular disease (0.9%). Other cardiovascular diseases accounted for 16.3% of the enrolled subjects (*n* = 18). Regarding metabolic comorbidities, diabetes mellitus' cohort prevalence was 9.09% (*n* = 10) while 19 patients presented dyslipidemia (17.2%) and the body mass index of 20 subjects resulted in obesity (18.1%). Finally, eight patients were affected by thyroiditis (7.27%) and 2 by chronic obstructive pulmonary disease (1.81%).

**Figure 1:**
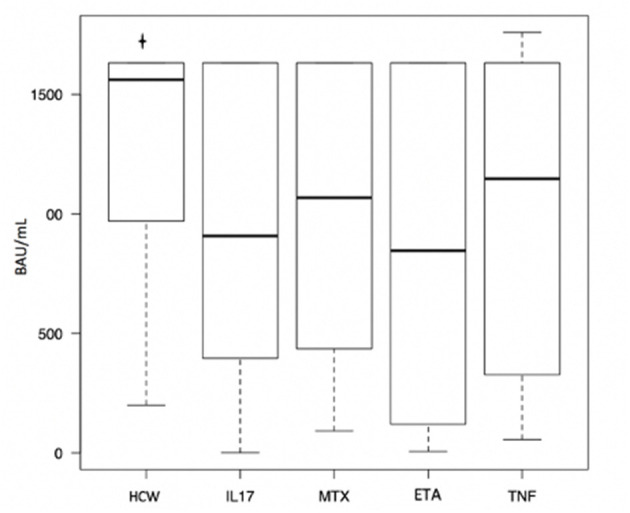
Differences in anti-SARS-CoV-2 Spike RBD antibody titers between psoriatic arthritis patients treated with different medications. HCW, HealthCare Workers control group; IL17, anti-IL17; MTX, methotrexate; ETA, etanercept; TNF, anti-TNF.

The control group (HCW) included 96 healthy health care workers. Of these, 31 (32.3%) were males and 65 were females (67.7%). The mean age of the control group was 50.54 years (SD 11.66). Mean disease activity calculated with DAPSA was 2.96 (SD 0.60) and it did not differ between patients undergoing different treatments (*p* = 0.779). Lymphocyte subpopulations (CD3+, CD3+/CD4+, CD3+/CD8+, CD3-/CD19+, CD3-/CD56+CD16+) did not show any differences between groups too (Table 1). All the PsA patients had a detectable humoral response, as well as for the subjects enrolled in HCW group. The median of anti-SARS-CoV-2 Spike RBD IgG antibodies levels in patients' cohort was 928.00 BAU/ml [IQR 329.25, 1632.0]. Considering the different groups, the median values were the following: 1068.00 BAU/ml [IQR 475.00, 1632.00] in patients under MTX therapy; 846.00 BAU/ml [IQR 125.00, 1632.00] in patients under etanercept treatment; 908.00 BAU/ml [IQR 396.00, 1632.00] in patients treated with anti-IL17 agents; 1148.00 BAU/ml [IQR 327.00, 1632.00] in patients under TNF-α inhibitors. No statistically significant differences were found between these groups (*p* = 0.73) (Table 2). The median serum level of HCW group was 1562.00 BAU/ml [IQR 975.00, 1632.00], significantly higher when compared to the patients' group (*p* ≤ 0.001) (Table 3; Figure 1). Linear regression analysis identified the age as negative predictor of concentration levels in the PsA population (β = −12.26, *p* = 0.016) but not in the control group.

**Table 1:** Differences in lymphocytes' subpopulations in the four subgroups.

**Treatment**	**Anti-IL17**	**Anti-TNF alpha**	**Etanercept**	**Metothrexate**	
***N***	**37**	**37**	**26**	**10**	***P*-value**
CD3 median (BAU/ml) [IQR]	1680.00 [1210.00, 1936.00]	1680.00 [1350.00, 1747.00]	1747.00 [1643.00, 1923.75]	1446.00 [1216.50, 1709.00]	0.4
CD4 median (BAU/ml) [IQR]	1073.00 [712.00, 1350.00]	1047.00 [709.00, 1350.00]	1087.00 [887.50, 1350.00]	894.50 [723.75, 1068.25]	0.498
CD8 median (BAU/ml) [IQR]	404.00 [290.00, 720.00]	414.00 [257.00, 764.00]	547.50 [306.25, 779.00]	556.50 [248.00, 738.25]	0.727
CD19 median (BAU/ml) [IQR]	204.00 [139.00, 275.00]	196.00 [139.00, 298.00]	225.00 [190.00, 311.50]	255.50 [144.75, 394.75]	0.520
CD56 median (BAU/ml) [IQR]	290.00 [166.00, 454.00]	370.00 [166.00, 527.00]	267.00 [178.75, 473.50]	268.50 [167.50, 324.50]	0.761

*BAU, binding antibody unit; IQR, inter-quartile range*.

**Table 2:** Differences in anti-SARS-CoV-2 Spike RBD antibody titers between psoriatic arthritis patients and HealthCare Workers control group.

	**Healthy healthcare controls workers**	**Treated PsA patients**	
***N***	**96**	**110**	***P*-value**
Anti-Spike IgG level median [IQR]	1562.00 [975.00, 1632.00]	928.00 [329.25, 1632.0]	0.000816

**Table 3:** Differences in anti-SARS-CoV-2 Spike RBD antibody titer in the four patient groups.

**Treatment**	**Anti-IL17**	**Anti-TNF alpha**	**Etanercept**	**MTX**	
***N***	**37**	**37**	**26**	**10**	***P*-value**
Anti-Spike IgG levels median [IQR]	908.00 [0.70, 1632.00]	1148.00 [56.00, 1760.00]	846.00 [6.40, 1632.00]	1068.00 [92.00, 1632.00]	ns

## Discussion

Recent recommendations indicate that patients with psoriatic disease who do not have contraindications to vaccination should receive an mRNA-based COVID-19 vaccine and they are invited to continue their systemic therapies for psoriasis and/or PsA in most cases (22).

Despite the lack of large studies focused on PsA populations, a reduction of the humoral response to SARS-CoV-2 vaccines in patients on immunomodulatory treatments has been repeatedly reported. Al-Janabi et al. recruited 120 participants with immune-mediated inflammatory diseases (IMIDs) in treatment with biologics, other immunomodulators or combination of therapy, reporting that 15% of patients with no prior COVID-19 failed to mount a detectable antibody response to BNT162b2 or AZD1222 vaccines while 41% had no detectable anti-SARS-CoV-2 Spike RBD S1 IgG antibodies. However, it is of note that the assessments were performed after a single dose of vaccine (23). Simon et al. enrolled 84 IMIDs patients and 182 controls, with no previous history of COVID-19, finding delayed and reduced overall responses to first or second vaccination dose in patients' group (13). Considering that patients under therapy did not show a different response compared with off-therapy patients, they concluded that the phenomenon may be related to the disease itself rather than the treatment. Geisen et al. evaluated antibody responses following the second dose of mRNA vaccines in 26 patients with IMIDs receiving biologic, conventional DMARDs and/or prednisolone compared to 42 healthy controls. They showed that all patients developed neutralizing antibodies, but mean levels of anti-S1 SARS-CoV-2 IgG titers were reduced in those under immunosuppression (15).

Similarly, we found lower antibody levels in response to vaccine in our PsA patients under immunomodulatory treatment, compared to the HCW group. In fact, the mean serum level in the PsA patients' group was 965.44 BAU/ml (SD 643.13) while it was significatively higher (*p* = 0.0000276) in the HCW group, being 1294.5 BAU/ml (SD 416.2). However, our cohort resulted in 100% seroconversion rate among patients receiving immunosuppression, as well as for healthy controls. Although reduced, detectable serological responses to vaccine in patients' group suggests successful induction of antibodies in individuals receiving methotrexate and targeted biologics.

While subgroup analysis of drug type could not be conducted (23) among the cited studies, Deepak et al. evaluated the titers of serum anti-SARS-CoV-2 spike (S) IgG in 133 adults with chronic inflammatory diseases, highlighting that patients on B-cell depletion therapy, prednisone, JAK inhibitors, and antimetabolites had statistically significant reductions in antibody titers in univariate and multivariate models. In contrast, antimalarials (i.e., hydroxychloroquine) and TNF-α inhibitors were not significantly associated with reduced antibody titers. However, most patients were able to mount an efficient immunological response to vaccine, while the highest rate of failed seroconversion was registered among patients under systemic corticosteroids or B-cell depleting therapies (24). In fact, rituximab and prednisone or prednisolone doses greater than 10 mg/die were also associated with higher risk of COVID-19 and hospitalization in patients with autoimmune or rheumatic diseases (25, 26), thus underlining the fundamental role of humoral response against SARS-CoV-2 infection.

In our case, when comparing single therapeutic regimens, no statistical difference emerged between different treatment groups.

Geisen et al. did not report significant differences in antibody levels comparing the according age groups (15); in our work, linear regression analysis revealed age as a negative predictor of anti-SARS-CoV-2 Spike RBD IgG levels in patients' cohort, while the same association was not found in HCW group. So, even if the significative difference between the mean age of the two groups (61.72 years, SD 12 vs. 50.54 years, SD 11.66, *p* < 0.001) could represent a limit of the study, age seemed not to influence antibodies production in control groups, thus reducing the risk of bias. Therefore, we may speculate that immunosuppressive agents could somehow enhance or anticipate the age-related immune senescence process in treated individuals. At this regard it is worth mentioning that a large cross-sectional study found a different age distribution of humoral response: in fact, the negative correlation with age was demonstrated only in people below the age of 18, while the adult population showed a positive correlation with higher antibodies titers in older age groups. Thus, other large-population studies are needed to improve the knowledge about this correlation (27).

For what concerns cellular response to vaccination, specific studies for psoriatic arthropathy are still lacking. However, a significant increase in Spike-specific B cells, T-follicular helper cells, activated CD4+ T cells and HLA-DR + CD8+ T cells was described using flow cytometry in IMIDs patients and controls, while activated CD8 + T cells and granzyme-B-producing CD8 + T cells boosting was lacking only in patients under methotrexate (16). Abatacept treatment in RA patients was also associated with reduced cellular T response, as well as with impaired production of neutralizing anti-Spike antibodies (28). With the aim to acquire more data on this topic, we evaluated lymphocyte subpopulations finding no differences between patients under different treatments. Moreover, lymphocyte subpopulations were not predictive of antibody levels according to linear regression analysis in our cohort. However, no decrease in any of the investigated subpopulations was observed, thus suggesting an adequate cellular immune response to vaccination.

To date, it is still not clear whether a reduced antibody response is invariably linked to an increased susceptibility to COVID-19. In fact, it has been reported that rates of SARS-CoV-2 infection appear to be similar between general population and patients with rheumatic diseases receiving DMARDs or biologics (29), including psoriasis (22, 30–35). In addition, those patients do not seem to have an increased risk of hospitalization or death from COVID-19, although generally burdened by higher rates of metabolic and cardiovascular comorbidities (36–40).

As already suggested by other authors (41), our results may support the decision not to suspend treatment with anti-TNF or anti-IL17 in the peri vaccination period. In fact, the latest version (4.0) of ACR guidelines Task Force failed to reach consensus on whether to temporarily interrupt these following each COVID vaccine dose, including both primary vaccination and supplemental (booster) dosing.

Among the limitations to our study immune functional tests such as plasma neutralization assay and assessment of interferon-γ produced by T-cells in response to SARS-CoV-2 peptides were not performed. Moreover, methotrexate was administered at a mean dosage of 10 mg/week. This may be not fully representative of PsA patients on methotrexate, which are often treated with higher dosages. Hence, humoral response to vaccination for PsA patients on methotrexate may not be comparable with the other drugs, despite our findings. The study did not cover all the treatment commonly used for psoriatic arthritis, i.e., abatacept, anti-IL23 and apremilast. In addition, the difference on mean age of the groups may represent a confounding factor.

While contributing to acquire more data concerning antibody response to vaccination on immunomodulatory treatments, our results do not exclude that antibody serum level may have been reduced by the disease itself rather than the treatment, as previously suggested by Simon et al. In fact, since the majority of patients underwent vaccination, as strongly recommended by the scientific community, adding a non-vaccinated PsA patients control group to the study was not possible.

## Conclusion

Our data show that systemic therapy for psoriatic arthritis, as observed for other rheumatic diseases, may lead to a reduced quantitative humoral response when compared with healthy controls. However, global seroconversion rate seems not to be significantly affected. There seem not to be statistically significant differences between the groups treated with low dose methotrexate and biologic agents with different mechanisms of action in terms of humoral response. Antibodies production may decrease with age, while immunosuppression could represent an enhancement for this phenomenon.

As we believe that cellular response might have a fundamental role into the development of immune response against SARS-CoV-2, further studies are needed to identify reliable indicators of its involvement and to clarify whether immunomodulatory treatments may affect it and how.

## Data Availability Statement

The raw data supporting the conclusions of this article will be made available by the authors, without undue reservation.

## Ethics Statement

All patients gave their written informed consent based on the prospective nature of the study according to the Declaration of Helsinki and to the Italian legislation (Authorization of the Privacy Guarantor n.9, 12 December 2013). The Institutional Review Board, Health Director of the Florence Hospital, has examined and approved this research and the use of clinical and laboratory data of common clinical practice, in compliance with the Privacy Law, for clinical and scientific studies and publications.

## Author Contributions

MB, AD, MI, MM, BL, VG, MC, and FLG designed the study and drafted the manuscript. MB, AD, EM, and AC drafted the manuscript. CA, LQ, AV, and EA revised the manuscript. All authors contributed to the article and approved the submitted version.

## Conflict of Interest

The authors declare that the research was conducted in the absence of any commercial or financial relationships that could be construed as a potential conflict of interest.

## Publisher's Note

All claims expressed in this article are solely those of the authors and do not necessarily represent those of their affiliated organizations, or those of the publisher, the editors and the reviewers. Any product that may be evaluated in this article, or claim that may be made by its manufacturer, is not guaranteed or endorsed by the publisher.
